# Pathology characteristics that optimize outcome prediction of a breast screening trial

**DOI:** 10.1054/bjoc.2000.1286

**Published:** 2000-07-24

**Authors:** T J Anderson, F E Alexander, J Lamb, A Smith, A P M Forrest

**Affiliations:** Departments of Pathology, Public Health Sciences, (Professor Emeritus) Clinical and Surgical Sciences, University of Edinburgh

**Keywords:** breast cancer, screening, pathology, prognosis, surrogate measure

## Abstract

The ability of pathology characteristics to predict outcome was tested with the 1029 cancers accumulated in the Edinburgh Randomized Trial of breast screening after 14 years follow-up. The majority (55.7%) were in the screening arm, which also had more operable cases (81.3% vs 62.2%); the reduction in the proportion of inoperable breast cancers in a UK female population invited to mammographic screening is a notable effect of the trial. In the 691 operable invasive cases the size, histological type, grade, node status and node number group individually showed highly significant (*P*< 0.001) association with survival. In multivariate analysis the Nottingham Prognostic Index (NPI) derived from these features showed highly significant association with survival (*P*< 0.001). However, when first adjusted for NPI, combined addition of pathological size in 6 categories and histological type as special or not had an independent association with survival that was statistically firmly based (*P*< 0.001). For operable breast cancer the gains are in smaller sizes, better histological features, and higher proportion node negative. The weighting factors applied to pathology indicators of survival in the NPI are not optimal for a population included in a trial of screening. In particular, a linear trend of the index with pathological size is not appropriate. Inclusion of histological type as special or not improves the index further. © 2000 Cancer Research Campaign


British Journal of Cancer (2000) 83(4), 487–492
© 2000 Cancer Research Campaign
doi: 10.1054/ bjoc.2000.1286, available online at http://www.idealibrary.com on IDEAL®

### Pathology characteristics that optimize outcome prediction of a breast screening trial

**TJ Anderson[1], FE Alexander[2], J Lamb[1], A Smith[2] and APM Forrest[3]**

[1]Departments of Pathology, [2]Public Health Sciences and [3](Professor Emeritus) Clinical and Surgical Sciences, University of Edinburgh

**Summary** The ability of pathology characteristics to predict outcome was tested with the 1029 cancers accumulated in the Edinburgh Randomized Trial of breast screening after 14 years follow-up. The majority (55.7%) were in the screening arm, which also had more operable cases (81.3% vs 62.2%); the reduction in the proportion of inoperable breast cancers in a UK female population invited to mammographic screening is a notable effect of the trial. In the 691 operable invasive cases the size, histological type, grade, node status and node number group individually showed highly significant ($P$ < 0.001) association with survival. In multivariate analysis the Nottingham Prognostic Index (NPI) derived from these features showed highly significant association with survival ($P$ < 0.001). However, when first adjusted for NPI, combined addition of pathological size in 6 categories and histological type as special or not had an independent association with survival that was statistically firmly based ($P$ < 0.001). For operable breast cancer the gains are in smaller sizes, better histological features, and higher proportion node negative. The weighting factors applied to pathology indicators of survival in the NPI are not optimal for a population included in a trial of screening. In particular, a linear trend of the index with pathological size is not appropriate. Inclusion of histological type as special or not improves the index further. © 2000 Cancer Research Campaign

**Keywords:** breast cancer; screening; pathology; prognosis; surrogate measure

The role of pathology characteristics of breast cancers as prognostic or predictive factors in management of breast cancer is widely acknowledged with recent editions of one leading journal devoted to this topic (Gasparini, 1998). The combination of histological features in the form of a single quantitative index of prognosis has been used for a number of functions. These include guidance on the type and extent of therapy, stratification for clinical trials, informal monitoring of screening programmes, and in the formal prediction of mortality differences in trials of screening or prevention (Day and Duffy, 1996).

The Nottingham Prognostic Index (NPI) was derived from a database of pathology and other characteristics (Haybittle et al, 1982), later assessed prospectively (Todd et al, 1987), and extended (Galea et al, 1992) to cover over 1600 cases. Use of the index in patients with symptomatic and operable disease has been independently validated (Brown et al, 1993; Balslev et al, 1994). However, although it is currently being used to predict mortality differences between the two arms of the UK trial of mammographic screening frequency it has not been validated in a screened population. The index relies on pathological size, axillary node status and histological grade, with size included as a linear term. It follows that each millimetre increase in size has the same effect on the value of the index, which may not be logical in light of the excess distribution of small cancers detected through screening. The size groupings used in assessment of the Two Counties Breast Screening Trial (Tabar et al, 1985) was distinctive at 1–9, 10–14, 15–19, 20–29, 30–49 and ≥50 mm, taking account of this changed distribution, may be more appropriate to the screening situation, and have been used in this study.

In previous reports we have emphasized the importance of histological special type as an independent determinant of survival in symptomatic (Dixon et al, 1985) and screen detected cancers (Anderson et al, 1986, 1991) and in this study the feature has been included as an additional variable.

The present report uses data from breast cancers diagnosed in women invited to participate in the Edinburgh Randomized Trial (ERT) of breast cancer screening. Such cancers have been systematically ascertained through a follow-up period of up to 14 years from the date of recruitment (1978–1985) and the outcome of mortality benefit reported (Alexander et al, 1999). The objective of this study was to identify criteria for inclusion in an optimal prognostic/predictive index for use in populations of screened women that might also be applicable to predicting mortality in screening trials.

*Received 24 January 2000*
*Revised 22 March 2000*
*Accepted 17 April 2000*

*Correspondence to:* TJ Anderson, Department of Pathology, Western General Hospital, Crewe Road, Edinburgh EH4 2XU, UK. E-mail:T.J.Anderson@ed.ac.uk

### METHODS

### Study population

The composition of the study population, the screening procedure, and the establishment of a pathology register have been described previously (Roberts et al, 1984). The women were invited for annual physical examination and biennial mammography over a period of time which depended on their year of randomization (8 years for those randomized 1978–81, 6 years for those in 1982–3, and 4 years for those in 1984–5). This period of intervention ended for all entrants around 1988 when NHS service screening became available to all women aged under 65 years. Breast cancers diagnosed up to 10 years from entry to the trial are included in the present analysis.

### Pathology evaluations

Entry to the database of pathology information relating to size, histological nature of cancers and lymph node status was through completion of a standardized form (Roberts et al, 1984). Slides of all cancers and axillary nodes were examined by two pathologists (TJA, JL) during the trial field work, including those diagnosed in other local pathology departments (Western General Hospital, St John's Hospital). In a few cases treated in distant hospitals, relevant data were extracted from pathology reports (TJA). The criteria for histological typing as 'special type' (ST) have been described (Anderson et al, 1986, 1991; Page and Anderson, 1987) and are in agreement with the National Coordinating Group (1995). Combined histological grade of cancers was assessed according to Elston and Ellis (1991) and criteria defined by the National Coordinating Group (1995). This was completed by the same pathologists (TJA, JL) in a separate review (blinded to detection method) of the relevant original slides. The consistency of grading between the reviewers was established on a subset of 52 cancers double read, giving kappa value of 0.54.

The customary size categories for invasive breast cancers are based on TNM (International Union Against Cancer, 1997) but in reports of the Two Counties breast screening trial (Tabar et al, 1985, 1992) the discrimination points were based on mm diameters less than 10, less than 15, and less than 20. In the present study we have considered 6 size categories (sizegroup), as 1 = 1–9, 2 = 10–14, 3 = 15–19, 4 = 20–29, 5 = 30–49 and 6 = ≥50 mm, as used by others (Day et al, 1989; Duffy et al, 1991), and are an alternative to measured size expressed as a linear variable, as used in NPI. The majority of patients had axillary node status determined by a node sample, which in the Edinburgh Breast Unit requires the removal of 4 nodes from the lower axillary fat contiguous with the axillary tail of the breast (Steele et al, 1985; Forrest et al, 1995). However, in general surgical practice both fewer or more nodes may be removed during the procedure. A smaller number of women had a total node dissection of all three levels of the axilla, which clearly provides a larger number of nodes for examination. On the standard record form the number of positive and negative nodes examined pathologically were separately entered, allowing the total number removed to be ascertained. We considered limiting information concerning the number of involved axillary nodes to examples with 4 or more nodes removed, but elected to include all dissections. We appreciate the limitations of a sampling procedure to determine the actual number of positive nodes, but this represents surgical practice as audited in Scotland (Scottish Cancer Therapy Network, 1996) and likely to be performed elsewhere in UK. In this paper 'node status' is either positive or negative, and 'node group' defines positivity as 1 = none, 2 = 1–3 and 3 = 4 or more involved.

The Nottingham Prognostic Index (= 0.2 × size [cm] + grade [1–3] + nodes [1–3], where 1 = node negative, 2 = 1–3 nodes positive, and 3 = >3 nodes or the apical node positive) was calculated from the available information. We have ignored information on apical node status, which was available only when an axillary clearance was performed. This formulation of axillary node information is suggested as a modification (Galea et al, 1992) of the original triple biopsy used in Nottingham, to conform with usual surgical practice; the NPI is now compiled from a sampling procedure similar to that in Edinburgh. In order to estimate the NPI where data was missing, multiple regression analysis was applied to the data set for which all the data was known; the dependent variable was the NPI and the independent variables were successive combinations of two and of one of the pathological characteristics. When detection group (see below) improved the model significantly it was included. This procedure yielded regression equations from which the NPI could be estimated for cases with missing data.

### Statistics

The cases forming the group for detailed study were those classified as operable invasive (i.e. lacking indication at diagnosis of being locally advanced and/or metastatic) and for whom at least one of the following characteristics was entered on the database:

- pathology size
- histological type
- combined histological grade
- node status and number positive.

Cox's proportional hazards method (Cox, 1972) was applied to investigate the relationship of pathology characteristics to the survival of cases from the time of diagnosis. Survival was censored if breast cancer was neither the underlying nor a contributory cause of death and censoring was applied at 14 years from trial entry date (or earlier in the case of women who entered the trial 1982–85 (see Alexander et al, 1999)). Since lead time bias and length biased sampling influence the survival from the time of diagnosis of screen-detected cancers, all cancers have been placed in one of four 'detection groups'; (i) prevalence (first) screen; (ii) incident (later) screen; (iii) interval cases and (iv) others. Adjustment for those detection groups has been applied systematically in the analyses.

Except where an alternative has been stated, all analyses of pathological size consider linear trend across six groups (sizegroup, see above), all of grade and node trend across three groups.

### RESULTS

### Population distribution of breast cancer severity

The distribution of 1029 cancers accumulated in the study, according to severity of disease state, as either in-situ, invasive operable or non-operable at diagnosis, is shown in Table 1. The two notable features are the excess of cancers in the screened population (55.7%), and the major proportion of inoperable cancers in the control population (36.8%), almost double that in those offered screening. The following analyses are restricted to 691 operable cases for which at least one item from the pathology data-set (size, type, grade, node positivity) was available. Several analyses are restricted to 458 cases for whom all of these were known. Of the 691 cases, 116 were from prevalence screen, 163 at incident screen, 64 were interval cases and 348 were 'other' detections (including those of the control arm of the trial).

### Interrelationships of size with histological type, grade and node positivity

As is shown in Figure 1 there are clear cut trends for higher grade with increasing size (expressed in six categories), and a similar trend is present for proportion by node group in Figure 2 ($P < 0.001$). A linear relationship with special histological type is less evident (Figure 3).

*© 2000 Cancer Research Campaign*

**Table 1** ERT cancers[1] by severity

| Trial Arm | Non-inv Micro-inv | Invasive Operable | Advanced or Mets | Total |
|---|---|---|---|---|
| Screening | 50 (8.7) | 416 (72.6) | 107 (19.7) | 573 (100) |
| Control | 13 (2.9) | 275 (60.3) | 168 (36.8) | 456 (100) |

[1]Number and (percentage)

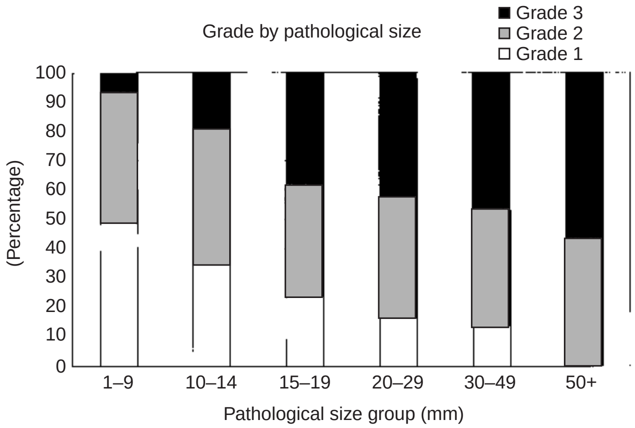

**Figure 1** Percentage distribution of invasive cancer grades within each of the incremental size categories

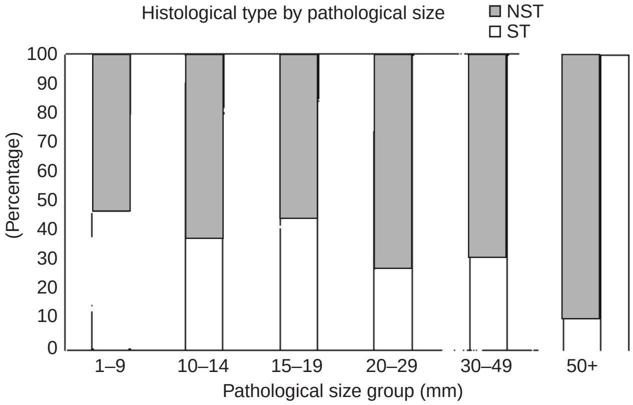

**Figure 3** Percentage distribution of node positive status (and number category) within each of the incremental size categories

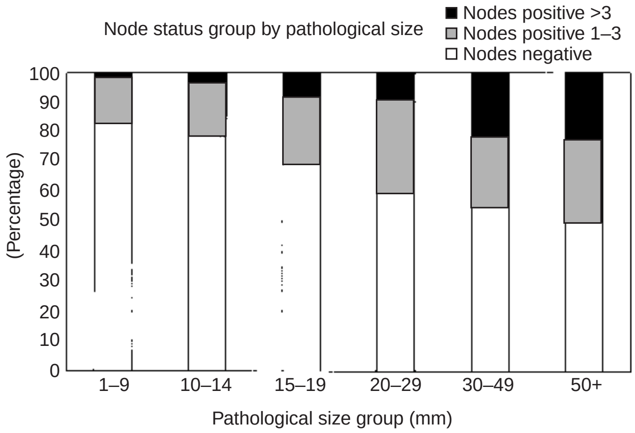

**Figure 2** Proportion of special type invasive cancers within each of the incremental size categories

### Analysis of pathological size

These analyses were restricted to the 672 cases for whom pathological size was known. Size was entered into the Cox regression model in two ways: (i) as actual size so that the model considered the linear effect of size and (ii) as the sizegroups described above. The addition of actual size as a linear trend to a model containing sizegroup was without effect (hazard ratio [HR] = 1.00, 95% CI 0.96–1.03 with adjustment for detection node group, and 0.97–1.03 without). On the other hand, when sizegroup was added to a model containing actual size, the HRs were 1.67 and 1.71 with 95% CI of 1.19–2.35 and 1.22–2.39, with and without adjustment for nodegroup respectively. The *P* values for inclusion of the extra terms in the model were 0.002 and 0.001. These data demonstrate that the use of the chosen six sizegroups is significantly more effective as a prognostic indicator than actual sizes expressed as a linear trend. The use of log (actual size + 0.5) was evaluated and was only slightly less effective than sizegroup.

### Univariate analysis of pathology characteristics

Univariate analysis of relevance for each pathological characteristic against survival, with size entered in six categories, grade as one of three classes, histological type as ‘special’ or ‘not special’, and node as status or group shows each to be highly significant (Table 2). The table also shows the number of cases for which each characteristic was available.

### Performance of the NPI

In these data the NPI is significantly associated with survival with and without adjustment for detection group (HR = 1.86, CI: 1.63–2.13; HR = 1.82, CI: 1.59–2.01, $P < 0.001$). However both size group and histological type, individually and in combination, make a further significant contribution to the survival model (Table 3). When considered by trial arm (Table 4), it is clear that the independent additional effect of size group applies particularly to the screening arm.

### Multivariate analysis of the Edinburgh data

When the pathology characteristics were tested in multivariate analysis, after adjustment for the others, they all contributed with independent significance (Table 5). Coefficients to apply in an index of prognosis are given in the table. These refer to the 691 operable cases, but similar results are given by the 458 with all

**Table 2** Univariate analyses[1] of pathology indicators

| Pathology Indicator | Number | HR | CI | *P*-value |
|---|---|---|---|---|
| Size group | 672 | 1.67 | 1.44 – 1.93 | <0.001 |
| Histological type | 631 | 2.96 | 1.85 – 4.74 | <0.001 |
| Grade | 586 | 2.37 | 1.80 – 3.11 | <0.001 |
| Node status | 629 | 2.99 | 2.08 – 4.29 | <0.001 |
| Node group | 629 | 2.19 | 1.75 – 2.73 | <0.001 |

[1]Analyses adjusted for detection group using Cox proportional hazard method.

**Table 3** Independent effect of pathology indicators after NPI adjustment

| Indicator[1] | Cases | HR | CI | *P*-value |
|---|---|---|---|---|
| Size group | 672 | 1.31 | 1.11 – 1.55 | 0.001 |
| Histological type | 583 | 1.81 | 1.10 – 2.94 | 0.015 |
| Grade | 586 | 1.26 | 0.87 – 1.83 | 0.21 |
| Node group | 629 | 0.91 | 0.63 – 1.39 | 0.5 |
| Size group | 574 | 1.31 | 1.10 – 1.56 | <0.001 |
| Histological type | | 1.92 | 1.16 – 3.17 | |

[1]Adjusted for detection group.

**Table 4** Independent effect of pathology indicators after NPI in each trial arm

| | Screening Arm | | | Control Arm | | |
|---|---|---|---|---|---|---|
| | HR | CI | P | HR | CI | P |
| Size group | 1.40 | 1.12 – 1.75 | 0.003 | 1.22 | 0.95 – 1.57 | 0.11 |
| Histological type | 1.69 | 0.91 – 3.15 | 0.08 | 2.02 | 0.89 – 4.58 | 0.07 |
| Size group | 1.42 | 1.12 – 1.79 | 0.003 | 1.17 | 0.91 – 1.52 | 0.07 |
| Histological type | 1.85 | 0.99 – 3.46 | | 2.17 | 0.90 – 5.22 | |

[1]All analyses of the screening arm adjust for cancer diagnosis group and NPI; all analyses of the control arm adjust for NPI.
[2]Analyses use all available data (ie all subjects with NPI and relevant additional indicators known).

**Table 5** Multivariate analysis and coefficients of the pathology indicators

| β | SE(β) | HR | CI | P[1] | |
|---|---|---|---|---|---|
| Size group[2] | 0.36 | 0.081 | (1.43) | (1.19 – 1.71) | <0.001 |
| Node group[3] | 0.54 | 0.12 | (1.97) | (1.31 – 2.96) | <0.001 |
| Grade | 0.53 | 0.15 | (1.74) | (1.24 – 2.46) | <0.001 |
| Histological type | 0.56 | 0.26 | (1.68) | (0.93 – 3.04) | 0.028 |

[1]For inclusion in the model in addition to all others in the Table
[2]Linear trends across the size groups: 1-9, 10-14, 15-19, 20-29, 30-49, 50+ mm.
[3]Linear trends across the three groups: 1=N neg., 2=1-3 N pos., 3= >3 N pos.
[4]Simplified index formula = 0.7 x size group + 1 x type + 1 x grade + 1 x node group.

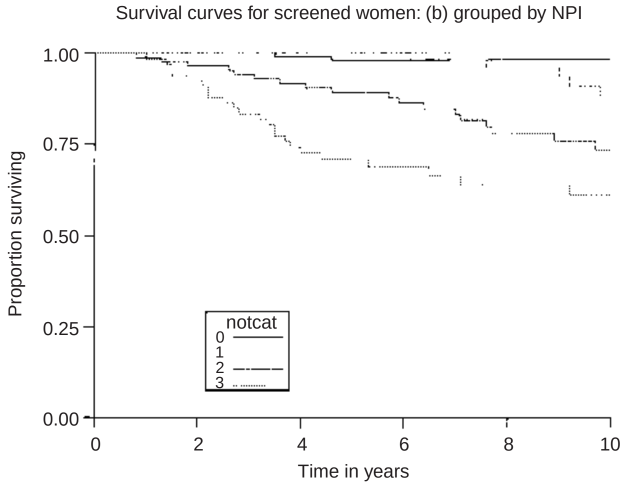

**Figure 4** Breast cancer survival curves of screened women divided into four groups (of equal numbers) according to Nottingham prognostic index (NOTCAT)

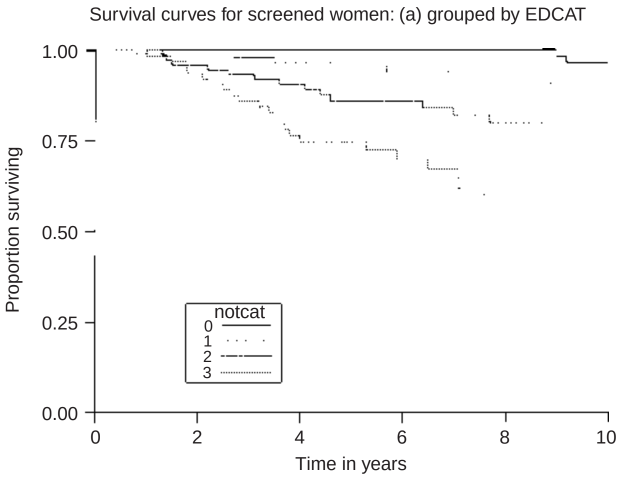

**Figure 5** Breast cancer survival curves of screened women divided into four groups (of equal numbers) according to the new index (EDCAT)

© 2000 Cancer Research Campaign data known. Applications of this index in comparisons with the NPI are shown in Figures 4 and 5. These figures are for screened women (i.e. prevalence screen detected, incidence screen detected and interval cases) and split cases into approximately four equal size groups for both NPI and the new index (NOTCAT, EDCAT).

When analyses were restricted to small cancers (<10 and 10–14 mm) the formula gave significant discrimination of benefit for 10–14 mm (HR 5.41, CI 1.77–16.58, $P$ = 0.008) but this was absent for cancers <10 mm alone.

### DISCUSSION

The pathological features of breast cancers in the two arms of the trial establish that screening in Edinburgh have two major effects. Firstly, the proportion of advanced or inoperable cases is almost halved, and secondly there is improvement in the proportion of invasive cancers of smaller size and node negative (Anderson et al, 1986, 1991). Both differences are acknowledged screening effects (Day et al, 1989) but the component of the mortality reduction attributable to each cannot be determined. Swedish studies report only a 10% proportion of advanced/inoperable disease (Frisell et al, 1987; Andersson et al, 1988) and allowance may need to be made for this fact in comparing screening effect between countries. However, the reductions in mortality at 14 years achieved in the ERT of 29% with censoring 3 years from end of trial field work (Alexander et al, 1999) are equivalent to those achieved in Sweden (Nystrom et al, 1993).

Breast cancer pathological features have also become accepted for inclusion into single indices, such as NPI, that in turn have allowed case classification into a small number (3–5) of separate prognostic categories. These may be put to different uses, but some relevant factors must first be acknowledged. The categories may be chosen to be of approximately equal numbers (as here) or selected by statistical criteria. If the latter, the choice of cut-point between two groups (good/poor prognosis) that minimizes the $P$ value for the comparisons between them (Altman et al, 1994; Sauerbrei et al, 1997) may over-estimate the effect (Beuttner et al, 1997); conversely, the equal numbers method ignores important data. It is pertinent that biostatisticians have used prognostic indices to predict future mortality for cases arising in randomized control trials of primary/secondary prevention (Day and Duffy, 1996) and that such predictions have smaller variance than observed mortality. It is thus essential that optimal use is made of prognostic data available at diagnosis. In this regard, the NPI has been used as the basis for comparisons of predicted mortality in women screened every three years or annually. Differences between cancers at the two intervals were small and did not achieve statistical significance (Duffy, personal communication). The current analysis has demonstrated that significant improvements are likely to be gained in the ability of pathology characteristics of operable invasive breast cancers to act as predictors for this outcome (surrogates) if they are entered according to particular size categories, as histological special type or not, by grade and also grouping by number of nodes with metastasis grouped as for the NPI.

The finding that the NPI, a widely accepted and clinically used measure to help determine therapy options, needs important modification to give optimal explanation of survival differences experienced with mammographic screening requires comment. It is clear that the effect of size is not linear, and that the regression coefficient employed in the NPI is not optimal for a population that contains a substantial proportion of mammographic screen-detected cases. A recent report of a Swedish population from 5 hospitals with similar characteristics also doubted the simple relationship with size (Sundquist et al, 1999). They used three size categories (≤10, 11–20, > 20 mm) and developed different factor coefficients, but endorsed the '…use of grade and the NPI in order to increase the comparability of groups of patients receiving different therapies'. It is therefore important to stress that the size groups chosen here give distinction between groups at all levels for grade and node groups (Figures 1, 2). The difference between categories 1 and 2, whilst only a few mm appears to be as important as that between 3 and 4 and 4 and 5.

The relevance of identifying special type cancers is accentuated in breast screening (Anderson et al, 1986, 1991; Tabar et al, 1996), on account of differing frequencies from symptomatic cases. Histological special type should not be interpreted as a 'competitive' factor to contrast with grade (Pereira et al, 1995). The two assessments are complementary in the sense that classical special type carries an importance for survival that is separable from grade. It is also likely that both histological type and grade are needed to interpret the natural history of breast cancer. Our data indicate a drift between size group, with larger size giving worse grade, and possibly more node spread and fewer special type. These issues have recently been discussed by Tabar et al (1999) and Anderson et al (2000).

The present findings indicate the need for re-evaluation of the pathology criteria used to measure screening effect, whether of trials or of service screening, as in the UK programmes. This applies both to use as surrogates of mortality benefit prediction or for setting targets for performance audit. This has particular relevance because of comments on outcome prediction for cancers <15 mm (Tabar et al, 2000). Our findings indicate that the available features discriminate well for cancers 10–14 mm, but confirm the need for supplementary data to identify the few cases with poor outcome in cancers <10 mm. We cannot address the question whether this is best achieved from additional pathology or radiology features. However, from an assessment of the ERT data base, the current data (potentially) available from UK screening pathology forms would be most informative when included as invasive cancer size in six categories and histological type as special or not, with histological grade and node metastasis in three groups each. A proposal for a formula is appended below Table 5. It is crucial to validate this model on an independent set of screening results with dedicated follow-up. It is hoped that one or other of the UK Trials set up to answer questions of mammographic screening feasibility, in terms of frequency, number of views or age at invitation, will provide the necessary data.

### ACKNOWLEDGEMENTS

We thank the medical and ancillary staff at the Breast Screening Clinic, staff members of the Radiological and Surgical Units at Longmore Hospital, and colleagues of the Department of Pathology at the University of Edinburgh and Western General Hospital. The trial was financed by a grant from the Chief Scientist's Office of the Scottish Home and Health Department to the Edinburgh Breast Screening Project (Committee members: Dr F Alexander, Dr TJ Anderson, Dr MM Andrew, Professor J Best, Dr C Brough, Mr U Chetty, Dr W Forbes, Professor APM Forrest (Chairman), Dr R Gruer, Dr A Huggins, Dr AE Kirkpatrick, Dr

© 2000 Cancer Research Campaign

NB Loudon, Mr W Lutz, Dr U Maclean, Dr MM Roberts (died 1989), and Mr J Duncan (project administrator). Jan Warner and staff members of the Information and Statistics Agency, and staff at the General Register Office, gave assistance with data collection. Grants from the Medical Research Council (G7803369, SPG8214335, TJA) and Cancer Research Campaign (SP1575, MMR) supported this work.

### REFERENCES

Alexander FE, Anderson TJ, Brown HK, Forrest APM, Kirkpatrick AE, Muir BB, Prescott RJ and Smith A (1999) 14 years of follow-up from the Edinburgh randomised trial of breast-cancer screening. *Lancet* **353:** 1903–1908

Altman DG, Lausen B, Sauerbrei W and Schumacher M (1994) Dangers of using 'optimal' cutpoints in the evaluation of prognostic factors. *J Natl Canc Inst* **86:** 829–835

Anderson TJ, Lamb J, Alexander FE, Lutz W, Chetty U, Forrest AP, Kirkpatrick A, Muir B, Roberts MM and Huggins A (1986) Comparative pathology of prevalence and incidence cancers detected by breast screening. *Lancet* **I:** 519–523

Anderson TJ, Lamb J, Doonan P, Alexander FE, Huggins A, Muir BB, Kirkpatrick AE, Chetty U, Hepburn W, Smith A, Prescott RJ and Forrest APM (1991) Comparative pathology of breast cancer in a randomised trial of screening. *Brit J Cancer* **64:** 108–113

Anderson TJ, Alexander FE, Forrest APM (2000) The natural history of breast cancer. What have we learned from screening? *Cancer* **87**: 1758–1759.

Andersson I, Aspergren K, Janzon L, Lundberg T, Lindholm K, Linell F, Ljungberg O, Ranstam J and Sigfusson B (1988) Mammographic screening and mortality from breast cancer: the Malmo mammographic screening trial. *Br Med J* 1988, **297:** 943–948

Balslev I, Axelsson CK, Zedeler K, Rasmussen BB, Carstensen B and Mouridsen HT (1994) The Nottingham Prognostic Index applied to 9149 patients from the studies of the Danish Breast Cancer Co-operative Group. *Breast Cancer Res Treat* **32:** 281–290

Beuttner P, Garbe C and Guggenmoos-Holzmann I (1997) Problems in defining cutoff points of continuous prognostic factors: example of tumor thickness in primary cutaneous melanoma. *J Clin Epidemiol* **50:** 1201–1210

Brown JM, Benson EA and Jones M (1993) Confirmation of a long-term prognostic index in breast cancer. *Breast* **2:** 144–147

Cox DR (1972) Regression models and life tables (with discussion). *J R Stat Soc* (B), **34:** 187–220

Day NE and Duffy SW (1996) Trial design based on surrogate end points: application to comparison of different breast screening frequencies. *J R Statist Soc A* **189:** 49–60

Day NE, Williams DRR and Khaw KT (1989) Breast cancer screening programmes: the development of a monitoring and evaluation system. *Brit J Cancer* **59:** 954–958

Dixon JM, Page DL, Anderson TJ, Lee D, Elton RA, Stewart HJ and Forrest APM (1985) Long-term survivors after breast cancer. *Br J Surg* **72:** 445–448

Duffy SW, Tabar L, Fagerberg G, Gad A, Grontoft O, South MC and Day NE (1991) Beast screening, prognostic factors and survival results from the Swedish two-county study. *Br J Cancer* **64:** 1133–1138

Elston CW and Ellis IO (1991) Pathological prognostic factors in breast cancer. I. The value of histological grade in breast cancer: experience from a large study with long term follow up. *Histopathol* **19:** 403–410

Forrest APM, Everington D, McDonald CC, Steele RJC, Chetty U and Stewart HJ (1995) The Edinburgh randomised trial of axillary node sampling or clearance after mastectomy. *Brit J Surg* 1995, **82:** 1504–1508

Frisell J, Eklund G, Hellstrom L and Somell A (1987) Analysis of interval breast carcinomas in a randomized screening trial in Stockholm. *Breast Cancer Res Treat* **9:** 219–225

Galea MH, Blamey RW, Elston CE and Ellis IO (1992) The Nottingham Prognostic Index in primary breast cancer. *Breast Cancer Res Treat* **22:** 207–219

Gasparini G (1998) Prognostic variables in node-negative and node-positive breast cancer – Part I and II. (Guest Editor) *Breast Cancer Res Treat* 51 (3) and 52 (1–3)

Haybittle JL, Blamey RW, Elston CW, Johnson J, Doyle P, Campbell FG, Nicholson RI and Griffiths K (1982) A prognostic index in primary breast cancer. *Br J Cancer* **45:** 361–366

International Union Against Cancer (UICC) (1997) TNM Classification of malignant tumours. LH Sobin and Ch. Wittekind (eds). 5th edition, Wiley-Liss: New York.

National Coordinating Group for Breast Screening Pathology (1995) Pathology reporting in breast cancer screening. NHSBSP publication no 3 (revised).

Nystrom L, Rutqvist LE, Wall S, Lindgren A, Lindqvist M, Ryder S, Andersson I, Bjurstam N, Fagerberg G, Frisell J, Tabar L and Larsson L-G (1993) Breast cancer screening with mammography: overview of Swedish randomised studies. *Lancet* **341:** 973–978

Page DL and Anderson TJ (1987) Diagnostic histopathology of the breast. pp 193–235. Churchill Livingstone, Edinburgh.

Pereira H, Pinder SE, Sibbering DM, Galea MH, Elston CW, Blamey RW, Robertson JFR and Ellis IO (1995) Pathological prognostic factors in breast cancer. IV. Should you be a typer or grader? A comparative study of two histological prognostic features in operable breast carcinoma. *Histopathol* **27:** 219–226

Roberts MM, Alexander FE, Anderson TJ, Forrest APM, Hepburn W, Huggins A, Kirkpatrick AE, Lamb J, Lutz W and MuirBB (1984) The Edinburgh randomised trial of screening for breast cancer: description of method. *Br J Cancer* **50:** 1–6

Sauerbrei W, Hubner K, Schmoor C and Schumacher M (1997) Validation of existing and development of new prognostic classification schemes in node negative breast cancer. *Breast Cancer Res Treat* **42:** 149–163

Scottish Cancer Therapy Network Scottish Breast Cancer audit 1987 & 1993 (1996) Report to Chief Scientist and CRAG. Information and Statistics Division, The National Health Service in Scotland.

Steele RJC, Forrest APM, Gibson T, Stewart HJ and Chetty U (1985) The efficacy of lowe axillary node sampling in obtaining axillary node status in breast cancer: a controlled randomised trial. *Brit J Surg* **72:** 368–369

Sundquist M, Thorstenson S, Brudin L, Nordenskjold B and the South East Swedish Breast Cancer Study Group (1999) Applying the Nottingham Prognostic Index to a Swedish breast cancer population. *Breast Cancer Res Treat* **53:** 1–8

Tabar L, Fagerberg CJ, Gad A, Baldetorp L, Holmberg LH, Grontoft O, Lunjguist U, Lundstrom B, Manson JC, Eklund G, Day NE and Pettersson F (1985) Reduction in mortality from breast cancer after mass screening with mammography. *Lancet* **I:** 829–832

Tabar L, Fagerberg G, Duffy SW, Day NE, Gad A and Grontoft O (1992) Update of the Swedish two county program of mammographic screening for breast cancer. *Radiol Clin North Amer* **30:** 187–210

Tabar L, Fagerberg G, Chen H-H, Duffy SW and Gad A (1996) Tumour development, histology and grade of breast cancers: prognosis and progression. *Int J Cancer* **66:** 413–419

Tabar L, Duffy SW, Vitak B, Chen H-H and Prevost TC (1999) The natural history of breast carcinoma. What have we learned from screening? *Cancer* **86:** 449–462

Tabar L, Chen H-H, Duffy SW, Yen MF, Chiang CF, Dean PB and Smith RA (2000) A novel method for prediction of long-term outcome of women with T1a, T1b, and 10–14 mm invasive breast cancers: a prospective study. *Lancet* **355:** 429–433

Todd JH, Dowie C, Williams MR, Elston CW, Ellis IO, Hinton CP, Blamey RW and Haybittle JL (1987) Confirmation of a prognostic index in primary breast cancer. *Br J Cancer* **56:** 489–492

*© 2000 Cancer Research Campaign*
